# The value of facial attractiveness for encouraging fruit and vegetable consumption: analyses from a randomized controlled trial

**DOI:** 10.1186/s12889-018-5202-6

**Published:** 2018-03-01

**Authors:** Katherine M. Appleton, Alanna J. McGrath, Michelle C. McKinley, Claire R. Draffin, Lesley L. Hamill, Ian S. Young, Jayne V. Woodside

**Affiliations:** 1 Research Centre for Behaviour Change, Department of Psychology, Bournemouth University, Poole House, Fern Barrow, Poole, BH12 5BB UK; 2 Centre for Public Health, School of Medicine, Dentistry and Biomedical Sciences, Queen’s University, Belfast, Grosvenor Road, Belfast, BT12 6BJ UK

**Keywords:** Fruit and vegetables, Facial attractiveness, Skin colour, Public health, Effect sizes

## Abstract

**Background:**

An effect of increased fruit and vegetable (FV) consumption on facial attractiveness has been proposed and recommended as a strategy to promote FV intakes, but no studies to date demonstrate a causal link between FV consumption and perceived attractiveness. This study investigated perceptions of attractiveness before and after the supervised consumption of 2, 5 or 8 FV portions/day for 4 weeks in 30 low FV consumers. Potential mechanisms for change via skin colour and perceived skin healthiness were also investigated.

**Methods:**

Faces were photographed at the start and end of the 4 week intervention in controlled conditions. Seventy-three independent individuals subsequently rated all 60 photographs in a randomized order, for facial attractiveness, facial skin yellowness, redness, healthiness, clarity, and symmetry.

**Results:**

Using clustered multiple regression, FV consumption over the previous 4 weeks had no direct effect on attractiveness, but, for female faces, some evidence was found for an indirect impact, via linear and non-linear changes in skin yellowness. Effect sizes, however, were small. No association between FV consumption and skin healthiness was found, but skin healthiness was associated with facial attractiveness.

**Conclusions:**

Controlled and objectively measured increases in FV consumption for 4 weeks resulted indirectly in increased attractiveness in females via increases in skin yellowness, but effects are small and gradually taper as FV consumption increases. Based on the effect sizes from this study, we are hesitant to recommend the use of facial attractiveness to encourage increased FV consumption.

**Trial registration:**

Clinical trial Registration Number NCT01591057 (www.clinicaltrials.gov). Registered: 27th April, 2012.

## Background

Fruit and vegetable (FV) consumption is associated with reduced risk from a number of major global health concerns [1, 2], including cardiovascular disease [3], stroke [4] and diabetes [5] (see [6, 7] for reviews). World Health Organization guidelines currently recommend the consumption of at least 400 g (five portions) of different fruit and vegetables a day for health benefits [1, 2], yet, it is commonly acknowledged that consumption levels in the UK, Europe and US fall below these recommendations [8–11]. In the UK, for example, adolescents, adults and older adults are reported to consume an average 180 g, 285, and 310 g FV / day respectively [9], and while averages for Europe are higher, wide differences between European countries are also reported [10]. Current strategies for increasing intakes [12–15], furthermore, are largely of limited impact [12–17].

One recent proposal considers the potential use of facial attractiveness for increasing FV intake [18, 19]. This hypothesis is based on suggestions that FV consumption contributes to skin colour, and that skin colour contributes to perceptions of healthiness and attractiveness [18, 19]. Improvements to appearance can be powerful motivators for undertaking health behaviours [20, 21], and several interventions targeting the appearance-related benefits of health behaviours have been successful [22–25]. For example, we found increased fruit consumption following exposure to an appearance- compared to a health-based health promotion poster [22], Jones and Leary [23] found increased safe sun behaviours after exposure to an essay on the effects of sun exposure on appearance compared with an essay on health risks or a control essay, Mahler et al. [24] found less skin darkening and more sun protective behaviours following provision of UV photographs and photoaging information compared with control, and Smith Klohn and Rogers [25] found improved intentions to undertake preventative behaviours following information on the disfiguring and visible nature of osteoporosis, compared to other types of information.

In relation to a hypothesis that FV consumption may increase facial attractiveness, studies are available in support of several individual steps. Firstly, FV consumption has been reported to contribute to skin colouration [26–29]. FV can be good sources of carotenoids [26, 27], many carotenoids are highly coloured (yellow-red) [26, 28, 29], and studies demonstrate detection of skin concentrations of carotenoids as a yellowness, slight redness and slight darkness to skin colour [26, 28, 29]. Relationships between FV consumption, skin concentrations of carotenoids and yellow skin have been reported [28–32], and increases in FV consumption have been associated with increases in skin yellowness, increases in skin redness and decreased skin lightness in prospective studies and controlled trials [33–35]. Secondly, associations between skin colour and perceptions of healthiness and attractiveness have also been reported [31, 35–39]. Facial images with high skin yellowness, high skin redness and low skin lightness have been rated as more healthy than images with reversed colouration, and participants increase the skin yellowness of computerized images to increase perceptions of skin healthiness [31, 36]. More healthy faces have also been reported as more attractive [37], and increased attractiveness has been associated with increased skin yellowness, increased skin redness and decreased skin lightness [35, 38, 39].

While the above evidence is consistent with the hypothesis, however, no studies thus far have reported a direct association between objectively measured FV consumption and attractiveness. No studies, furthermore, have investigated the impact on facial attractiveness of a deliberate increase in FV consumption. Before attractiveness-based interventions are developed, work is required to demonstrate that changes in FV consumption can result in changes in perceptions of attractiveness. This study investigated perceptions of attractiveness before and after a controlled and objectively measured increase in FV consumption for 4 weeks. To investigate proposed mechanisms, both a direct relationship and a relationship mediated by skin colour and perceptions of healthiness were considered.

## Methods

The work was undertaken as an addition to a study where FV consumption was prescribed and controlled for a 4 week period. Full details of the primary study are published elsewhere (trial registration: NCT01591057) [40]. In that study, 30 habitual low consumers of FV (habitually consuming less than or equal to 2 portions of FV/day at study entry) were randomized to receive 2, 5 or 8 FV portions/day for a 4 week period, where all foods were provided, consumption was supervised during two meals/day on weekdays, and all other consumption was verified. FV consumption thus increased for two groups of consumers (5 and 8 FV portions/day groups), but remained stable for the 2 FV portions/day group. The 2 FV portions/day group, thus, acted as a control group, to control for effects as a result of the provision of foods, due to inclusion in a trial and effects due to changes over the time period. Increases in FV consumption to 5 and 8 FV portions/day were used to reflect a recommended and a high FV consumption based on current UK recommendations. All participants were provided with a variety of fruit and vegetables based on their preferences and pre-study diet. A sample size of 10 participants per group was sufficient for the primary purpose of the work [40]. A 4 week intervention period was also dictated by the primary purpose of the study, but 4 weeks is adequate for changes in plasma/serum concentrations and skin concentrations of water-soluble and fat-soluble vitamins and other polyphenolic biomarkers of FV intake [27, 33, 34, 41]. At the start and end of the 4 week period, all participants were photographed in controlled conditions.

### Facial images

Facial photographs were gained from all 30 participants of the primary controlled feeding study.

Facial photographs were taken using a fixed tripod-mounted Fuji FinePix S5500 digital camera, in a room with no natural light and good artificial lighting. Lighting was from fluorescent tubes from above and the front such that shadows were not created on the face and reflections from clothing were minimal. For all photographs, participants were asked to wear a white headband obscuring their facial hairline, maintain an expressionless face and were seated facing the camera against a white background. Participants were asked to remove any self-tanning products and/or facial makeup prior to photography, and use of sunbathing, sunbeds and self-tanning products and time since last shave were recorded. None of the participants had substantial facial hair, e.g. beards. Three photographs were taken at each time point to ensure at least one suitable image was obtained from each participant, e.g. in case the eyes were closed. Images were judged for suitability in the order in which they were taken. Camera settings and placement, seating and room lighting remained constant and in place for the whole study period.

Prior to further use, suitable facial photographs were first checked against a MacBeth colourchecker to ensure broad colour agreements, and were compared using Adobe CS Photoshop to ensure against differences in background colour. The Light-dark (L*), red-green (a*) and yellow-blue (b*) values of five 5 mm-diameter spot checks of the background in each photograph were compared across all photographs, to ensure comparability in photograph colouration across FV groups and time points. Photographs were then cropped such that all background, including headband and hair, were blackened, to prevent distraction by these aspects of each photograph. Objective assessments of skin colour, and changes in skin colour over the 4 week period (L*, a*, b*) were also made using five 5 mm-diameter spot checks of the forehead of the face in each image. Spot check areas were selected randomly, while avoiding areas with skin blemishes and high reflective luminance.

### Facial image rating

All facial images were subsequently rated in an independent study for attractiveness, skin colour and healthiness. Participants for the rating study were recruited from the student population of Bournemouth University, UK, had normal vision, wore corrective equipment during the study where normally used, and were not knowingly colour-blind.

Facial images were rated using 100 mm visual analogue scales, anchored at both ends, for attractiveness (How attractive is this person?, not at all – extremely); skin yellowness (How yellow is this person’s skin?, not at all – extremely); skin redness (How red is this person’s skin?, not at all – extremely); and skin healthiness (How healthy is this person?, not at all – extremely). Ratings were also requested for skin clarity (How clear is this person’s skin?, not at all – extremely); and facial symmetry (How symmetrical is this person’s face?, not at all – extremely), as other known determinants of facial attractiveness, we thought unlikely to be affected by FV consumption [42, 43]. These types of measures are considered reliable, valid and desirable methods for assessing subjective perceptions [44]. Subjective ratings of skin colour, as well as objective measures, were made to enhance the ecological validity of the study. Appearance questions were also interspersed with questions on four personality characteristics – caring, friendly, honest, intelligent, as distractors.

Facial photographs were presented on a 24″ LED computer screen (BENQ XL 24″, 1920 × 1080 pixels, 60 Hz refresh rate, colour calibrated), one at a time, using a SuperLab presentation program. Photographs were presented for 20,000 ms interspersed with a 3000 ms blank screen, where order of presentation for all 60 photographs was randomized for each participant (randomization occurred across FV group and before/after FV consumption). The presentation set-up remained constant for all photographs and all participants throughout the study. Colour calibration of the presentation display was completed once at the start of the study and verified at the end. All ratings were completed while each photograph was displayed. Prior to study completion, all participants took part in a practice session using four photographs of the experimenter to familiarize themselves with the procedure and rating scales. Participants only proceeded to the study photographs once they were content to do so. Participants were offered breaks during testing after every 20 photographs to ensure against fatigue.

### Analysis

Analyses were conducted using clustered multiple regression, using rater ID as the cluster variable. Multiple regression was used to demonstrate effects of the intervention while controlling for baseline values [45], and clustered multiple regression allowed the consideration of all 60 assessments from each individual in all analyses, while any other analysis, e.g. analysis of variance, would have required some initial reduction of the data. Analyses were first conducted for the sample as a whole, and due to potential differences between ratings of male and female faces [36, 38, 43], analyses were also undertaken separately for male and female faces. In primary models, ratings of perceived attractiveness were predicted using FV consumption over the past 4 weeks, when controlling for face ID and baseline ratings of attractiveness. In secondary models, to test for mediation [46], ratings of attractiveness were predicted using ratings of skin yellowness, skin redness, skin healthiness, skin clarity and facial symmetry (when controlling for face ID and baseline variables); and ratings of skin yellowness, skin redness, skin healthiness, skin clarity and facial symmetry were predicted using FV consumption over the past 4 weeks (when controlling for face ID and baseline variables). Non-linear (squared) as well as linear relationships were investigated for all variables, using the same methods, due to possible non-linear relationships between skin colour and healthiness [47–50]. In final models, ratings of perceived attractiveness were predicted using FV consumption over the past 4 weeks, when controlling for face ID, baseline ratings of attractiveness and significant mediators. There were no missing data. All analyses were conducted in Stata (StataCorp Inc.). Results are presented as standardized co-efficients. Standardized coefficients for linear relationships demonstrate the change in the dependent variable as a function of its standard deviation, for every standard deviation change in the independent variable/s. Non-linear coefficients demonstrate the change in a linear relationship as the independent variable increases, to demonstrate an accelerating or decelerating linear relationship. Significance was set at *p* < 0.05.

## Results

### Facial images

Participants in the feeding study were 15 females, 15 males; mean age = 29 years, range = 19–61 years; all non-smokers. Demographic, various lifestyle characteristics (e.g. alcohol use, medication use) and various anthropometric measurements (e.g. BMI, blood pressure) were evenly distributed across FV consumption groups [40]. Twenty-seven participants were white Caucasian, three male participants were of Asian origin, again distributed across the three FV consumption groups. All participants adhered correctly to the full study protocol – all participants consumed the required FV per day based on laboratory observations, diaries and food returns. Plasma concentrations of Vitamin C and serum concentrations of polyphenolic FV intake markers (lutein, β-cryptoxanthin, α-carotene, β-carotene) also differentially increased in the three FV consumption groups over the 4 week period [40]. Lutein, β-cryptoxanthin, α-carotene and β-carotene increased in the 5 FV portions/day by 0.03 (95% CI: 0.004, 0.06) – 0.36 (95% CI: 0.05, 0.67) μmol/l and in the 8 FV portions/day group by 0.06 (95% CI: 0.01, 0.11) – 0.88 (95% CI: 0.31, 1.46) μmol/l, while changes in the 2 FV portions/day group ranged from − 0.01 (95% CI: -0.03, 0.01) – 0.14 (95% CI: 0.01, 0.26) μmol/l. (Please see the original paper [40] for full details.) All of these polyphenolic compounds are coloured yellow-red.

For all participants, the first photograph taken at each time point was used for the ratings study. Use of sunbathing, sunbeds and self-tanning products by participants, and time since last shave did not differ between time points. Light-dark (L*), red-green (a*) and yellow-blue (b*) values of the grey background for all photos were similar (L* CoV = 29%, a* CoV = 35%, b* CoV = 4%). Yellow-blue (b*) values of the foreheads of the faces were significantly higher (more yellow) in the groups consuming 5 and 8 FV portions/day after the 4 weeks compared to before (smallest t(8) = 2.63, *p* = 0.03), while no change was found in the group consuming 2 FV portions/day for 4 weeks (t(10) = 0.90, *p* = 0.39). Yellow-blue values were also higher for male compared to female faces (F(1) = 5.32, *p* = 0.03). There were no interactions between FV group and gender (F(2) = 0.14, *p* = 0.87), but numbers per group are very small. Light-dark (L*) values were also found to differ between genders (F(1) = 7.22, *p* = 0.01), where female faces were lighter, but no differences over time or by group were found (largest F(2) = 1.01, *p* = 0.38). No effects were found in red-green (a*) values (largest F(1) = 1.55, *p* = 0.23). L*, a* and b* values are given in Table 1.

**Table 1:** Mean (standard deviation (sd)) light-dark (L*), red-green (a*) and yellow-blue (b*) values for 5 spot checks on the foreheads of all facial images (*n* = 30)

FV group	Gender	L*		a*		b*	
		Before	After	Before	After	Before	After
2 FV portions/day	Male (*n* = 5)	61.5 (2.3)	62.5 (3.9)	10.9 (1.6)	10.7 (3.6)	21.3 (3.9)	22.5 (3.1)
	Females (*n* = 6)	66.9 (4.3)	68.1 (4.0)	8.2 (3.5)	8.3 (2.3)	19.7 (0.6)	19.6 (1.1)
5 FV portions/day	Male (*n* = 5)	62.7 (3.8)	62.2 (3.3)	9.1 (2.1)	8.5 (4.0)	21.0 (4.4)	23.0 (3.9)
	Females (*n* = 4)	67.1 (3.7)	67.3 (5.2)	9.3 (3.5)	8.8 (2.3)	18.1 (3.4)	20.9 (3.8)
8 FV portions/day	Male (*n* = 5)	62.7 (5.5)	61.6 (4.9)	11.7 (6.7)	13.2 (7.7)	22.3 (4.1)	24.5 (6.0)
	Females (*n* = 5)	64.3 (4.1)	62.1 (3.9)	9.6 (2.0)	9.5 (2.3)	19.0 (1.9)	20.3 (3.3)

### Facial image rating

Facial images were rated by 73 individuals - 33 males, 40 females, all white Caucasian (No further demographic details were recorded). Descriptive statistics for all ratings are shown in Table 2. Ratings for all characteristics ranged between 0 and 100 mm, with mean (standard deviation) ratings of all variables ranging between 31 (22) – 63 (18) mm.

**Table 2:** Mean (standard deviation (sd)) and range rating (mm) of each facial attribute for male (*N* = 15) and female (*N* = 15) facial images from all raters (*N* = 73)

	2 portions/day				5 portions/day				8 portions/day			
	Before		After		Before		After		Before		After	
	Mean (sd)	Range	Mean (sd)	Range	Mean (sd)	Range	Mean (sd)	Range	Mean (sd)	Range	Mean (sd)	Range
Male faces												
Attractiveness	33 (21)	0–84	33 (21)	0–81	31 (20)	0–91	32 (20)	0–90	38 (19)	2–91	35 (20)	0–87
Skin yellowness	35 (26)	0–100	39 (26)	0–100	38 (24)	0–94	38 (24)	0–100	41 (26)	0–100	42 (26)	0–100
Skin redness	34 (22)	0–89	36 (24)	0–97	37 (22)	0–100	38 (23)	0–100	33 (26)	0–100	38 (26)	0–100
Skin healthiness	52 (22)	0–96	49 (22)	0–95	47 (20)	0–97	49 (22)	0–100	58 (18)	4–100	53 (20)	2–100
Skin clarity	57 (20)	0–96	55 (21)	0–100	51 (24)	0–100	50 (24)	0–100	63 (18)	1–100	59 (20)	2–99
Facial symmetry	51 (21)	1–96	49 (23)	0–94	50 (21)	0–94	51 (21)	0–94	48 (22)	0–98	49 (21)	0–97
Female faces												
Attractiveness	38 (22)	0–93	36 (21)	0–90	37 (22)	0–95	34 (22)	0–87	33 (19)	0–92	34 (19)	1–92
Skin yellowness	34 (24)	0–91	35 (24)	0–93	31 (22)	0–90	36 (25)	0–97	36 (25)	0–98	41 (24)	0–95
Skin redness	37 (23)	0–100	39 (23)	0–100	44 (25)	0–95	40 (24)	0–89	34 (24)	0–96	34 (23)	0–89
Skin healthiness	54 (21)	0–100	50 (21)	0–100	53 (21)	5–100	51 (21)	0–100	50 (19)	0–100	50 (19)	0–100
Skin clarity	50 (26)	0–100	46 (27)	0–100	40 (28)	0–95	42 (27)	0–99	48 (23)	0–100	44 (21)	1–97
Facial symmetry	57 (20)	5–95	56 (20)	4–95	51 (20)	0–94	51 (22)	1–99	54 (21)	2–94	52 (19)	2–94

#### All facial images

Results from all regression analysis on all facial images are shown in Table 3. Initial analyses revealed no association between FV consumption over the last 4 weeks and attractiveness (β = 0.06, 95%CIs − 0.15, 0.26, *p* = 0.57). Analyses of relationships between FV consumption and possible mediators revealed positive associations between FV consumption over the past 4 weeks and skin yellowness (linear relationship: β = 0.49, 95%CIs 0.20, 0.78, *p* < 0.01; non-linear relationship: β = 36.3, 95%CIs 9.7, 62.9, *p* = 0.01). Analyses of relationships between possible mediators and perceived attractiveness demonstrated positive relationships between all mediators and attractiveness, excepting skin redness (smallest skin yellowness: linear relationship: β = 0.07, 95%CIs 0.02, 0.11, *p* < 0.01; non-linear relationship: β = < 0.01, 95%CIs < 0.01, < 0.01, *p* = 0.04). Significant mediators were thus, skin yellowness (linear and non-linear relationships). Final models included these mediators and revealed that greater attractiveness at 4 weeks was not directly associated with FV consumption over the previous 4 weeks (β = − 0.01, 95%CIs − 0.22, 0.20, *p* = 0.92), but was associated with skin yellowness (linear relationship: β = 0.25, 95%CIs 0.08, 0.42, *p* < 0.01; non-linear relationship (β = − < 0.01, 95%CIs - < 0.01, − < 0.01, *p* = 0.03).

**Table 3:** Standardized coefficients for all regression models investigating ratings of all facial attributes before and after 2 vs 5 vs 8 portions/day FV consumption over the past 4 weeks in all facial images*

	FV consumption – attractiveness^1^					
	β		95% CI		*p.*	
FV consumption	0.06		−0.15, 0.26		0.57	
	FV consumption – Mediating variables^2^			Mediating variables – Attractiveness^3^		
	β	95% CI	*p.*	β	95% CI	*p.*
Skin yellowness	**0.49**	**0.20, 0.78**	**< 0.01**	**0.07**	**0.02, 0.11**	**< 0.01**
Skin yellowness squared	**36.3**	**9.7, 62.9**	**0.01**	**< 0.01**	**< 0.01, < 0.01**	**0.04**
Skin redness	− 0.02	− 0.28, 0.24	0.86	0.03	− 0.01, 0.08	0.12
Skin redness squared	8.08	−14.6, 30.8	0.48	< 0.01	- < 0.01, < 0.01	0.36
Skin healthiness	0.17	−0.04, 0.39	0.13	**0.28**	**0.23, 0.33**	**< 0.01**
Skin healthiness squared	3.7	18.9, 26.3	0.74	**< 0.01**	**< 0.01, < 0.01**	**< 0.01**
Skin clarity	0.02	−0.26, 0.30	0.90	**0.16**	**0.12, 0.19**	**< 0.01**
Skin clarity squared	−17.8	−45.9, 10.3	0.21	**< 0.01**	**< 0.01, < 0.01**	**< 0.01**
Facial symmetry	−0.08	−0.34, 0.18	0.52	**0.18**	**0.13, 0.23**	**< 0.01**
Facial symmetry squared	−18.4	−45.1, 8.3	0.17	**< 0.01**	**< 0.01, < 0.01**	**< 0.01**
	FV consumption – attractiveness including mediators^4^					
	β		95% CI		*p.*	
FV consumption	−0.01		− 0.22, 0.20		0.92	
Skin yellowness	**0.25**		**0.08, 0.42**		**< 0.01**	
Skin yellowness squared	**- < 0.01**		**- < 0.01, − < 0.01**		**0.03**	
Baseline attractiveness	**0.65**		**0.60, 0.70,**		**< 0.01**	

*Facial images were gained in Belfast, UK, 2011. Ratings of facial attributes were gained in Bournemouth, UK, 2013–2015

Significant relationships (*p <* 0.05) are emboldened

^1^Primary models - ratings of perceived attractiveness were predicted using FV consumption over the past 4 weeks, when controlling for face ID and baseline ratings of attractiveness. Overall model performance *R*^2^=0.47, F(3,72) = 310.37, *p* < 0.01

^2^Secondary models - ratings of skin yellowness, skin redness, skin healthiness, skin clarity and facial symmetry were predicted using FV consumption over the past 4 weeks (when controlling for face ID and baseline variables)

^3^Secondary models - ratings of attractiveness were predicted using ratings of skin yellowness, skin redness, skin healthiness, skin clarity and facial symmetry (when controlling for face ID and baseline variables);

^4^Final models - ratings of perceived attractiveness were predicted using FV consumption over the past 4 weeks, when controlling for face ID, baseline ratings of attractiveness and significant mediators. Overall model performance *R*^2^=0.48, F(5,72) = 278.82, *p* < 0.01

In these models, increased FV consumption by a standard deviation of 2.5 FV portions/day resulted in an increase in skin yellowness (based on a standard deviation of 25 mm (Table 2)) of approximately 12.5/100 mm, and a change in skin yellowness of approximately 12.5 mm results in an increase in attractiveness (based on a standard deviation of 20 mm (Table 2)) of approximately 2.5/100 mm. The significant non-linear term suggests that increases in skin yellowness and attractiveness decrease as FV consumption and skin yellowness increase respectively, thus as more FV is consumed, impacts on skin yellowness reduce, and similarly, as skin yellowness increases, impacts on attractiveness reduce.

#### Males

Results from all regression analysis on male facial images are shown in Table 4. Initial analyses revealed no association between FV consumption over the last 4 weeks and attractiveness (β = − 0.17, 95%CIs − 0.45, 0.11, *p* = 0.24). Analyses of relationships between FV consumption and possible mediators revealed positive associations between FV consumption over the past 4 weeks and skin redness (non-linear relationship: β = 50.6, 95%CIs 15.1, 86.1, *p* < 0.01). Analyses of relationships between possible mediators and perceived attractiveness revealed that increased skin yellowness (linear relationship only), skin healthiness, skin clarity and facial symmetry were associated with increased attractiveness (smallest: skin yellowness: β = 0.06, 95%CIs 0.01, 0.11, *p* = 0.02). None of the mediators were significantly associated with both FV consumption and attractiveness. Final models were identical to initial models - greater attractiveness at 4 weeks was not associated with FV consumption (β = − 0.17, 95%CIs − 0.45, 0.11, *p* = 0.24).

**Table 4:** Standardized coefficients for all regression models investigating ratings of all facial attributes before and after 2 vs 5 vs 8 portions/day FV consumption over the past 4 weeks in male facial images*

	FV consumption – attractiveness^1^					
	β		95% CI		*p.*	
FV consumption	−0.17		−0.45, 0.11		0.24	
	FV consumption – Mediating variables^2^			Mediating variables – Attractiveness^3^		
	β	95% CI	*p.*	β	95% CI	*p.*
Skin yellowness	0.32	−0.12, 0.76	0.15	**0.06**	**0.01, 0.11**	**0.02**
Skin yellowness squared	23.5	−22.1, 69.2	0.31	< 0.01	- < 0.01, < 0.01	0.19
Skin redness	0.35	−0.03, 0.73	0.07	0.01	−0.03, 0.06	0.60
Skin redness squared	**50.6**	**15.1, 86.1**	**< 0.01**	< 0.01	- < 0.01, < 0.01	0.98
Skin healthiness	−0.03	−0.35, 0.29	0.84	**0.29**	**0.23, 0.35**	**< 0.01**
Skin healthiness squared	−12.6	−42.1, 16.9	0.40	**< 0.01**	**< 0.01, < 0.01**	**< 0.01**
Skin clarity	−0.02	−0.41, 0.36	0.91	**0.16**	**0.11, 0.22**	**< 0.01**
Skin clarity squared	2.06	−36.3, 40.4	0.92	**< 0.01**	**< 0.01, < 0.01**	**< 0.01**
Facial symmetry	0.29	−0.09, 0.68	0.14	**0.17**	**0.11, 0.23**	**< 0.01**
Facial symmetry squared	18.8	−22.1, 59.7	0.36	**< 0.01**	**< 0.01, < 0.01**	**< 0.01**
	FV consumption – attractiveness including no mediators^4^					
	β		95% CI		*p.*	
FV consumption	−0.17		− 0.45, 0.11		0.24	
Baseline attractiveness	**0.70**		**0.64, 0.75,**		**< 0.01**	

*Facial images were gained in Belfast, UK, 2011. Ratings of facial attributes were gained in Bournemouth, UK, 2013–2015

Significant relationships *(p < *0.05) are emboldened

^1^Primary models - ratings of perceived attractiveness were predicted using FV consumption over the past 4 weeks, when controlling for face ID and baseline ratings of attractiveness. Overall model performance *R*^2^=0.48, F(3,72) = 210.96, *p* < 0.01

^2^Secondary models - ratings of skin yellowness, skin redness, skin healthiness, skin clarity and facial symmetry were predicted using FV consumption over the past 4 weeks (when controlling for face ID and baseline variables)

^3^Secondary models - ratings of attractiveness were predicted using ratings of skin yellowness, skin redness, skin healthiness, skin clarity and facial symmetry (when controlling for face ID and baseline variables);

^4^Final models - ratings of perceived attractiveness were predicted using FV consumption over the past 4 weeks, when controlling for face ID, baseline ratings of attractiveness and significant mediators. Overall model performance *R*^2^=0.48, F(3,72) = 210.96, *p* < 0.01

#### Females

Results from all regression analysis on female facial images are shown in Table 5. Initial analyses revealed no association between FV consumption over the last 4 weeks and attractiveness (β = 0.15, 95%CIs − 0.20, 0.49, *p* = 0.39). Analyses of relationships between FV consumption and possible mediators revealed positive associations between FV consumption over the past 4 weeks and skin yellowness, and negative relationships between FV consumption and skin redness and facial symmetry (smallest: facial symmetry: linear relationship: β = − 0.56, 95%CIs − 0.93, − 0.19, *p* < 0.01; non-linear relationship: β = − 66.7, 95%CIs − 103.2, − 30.2, *p* < 0.01). Analyses of relationships between possible mediators and perceived attractiveness demonstrated positive relationships between all mediators and attractiveness, excepting skin redness (smallest skin yellowness: linear relationship: β = 0.08, 95%CIs 0.02, 0.13, *p* = 0.01; non-linear relationship: β = < 0.01, 95%CIs < 0.01, < 0.01, *p* = 0.03). Significant mediators were thus, skin yellowness (linear and non-linear relationships) and facial symmetry (linear and non-linear relationships). Final models included these mediators and revealed that greater attractiveness at 4 weeks was not directly associated with FV consumption over the previous 4 weeks (β = 0.16, 95%CIs − 0.20, 0.52, *p* = 0.38), but was associated with skin yellowness (linear relationship: β = 0.24, 95%CIs 0.10, 0.38, *p* < 0.01; non-linear relationship (β = − < 0.01, 95%CIs - < 0.01, − < 0.01, *p* < 0.01).

**Table 5:** Standardized coefficients for all regression models investigating ratings of all facial attributes before and after 2 vs 5 vs 8 portions/day FV consumption over the past 4 weeks in female facial images *

	FV consumption – attractiveness^1^					
	β		95% CI		*p.*	
FV consumption	0.15		−0.20, 0.49		0.39	
	FV consumption – Mediating variables^2^			Mediating variables – Attractiveness^3^		
	β	95% CI	*p.*	β	95% CI	*p.*
Skin yellowness	**0.93**	**0.48, 1.39**	**< 0.01**	**0.08**	**0.02, 0.13**	**0.01**
Skin yellowness squared	**73.4**	**33.3, 113.4**	**< 0.01**	**< 0.01**	**< 0.01, < 0.01**	**0.03**
Skin redness	**−0.74**	**−1.13, − 0.36**	**< 0.01**	0.05	− 0.01, 0.11	0.08
Skin redness squared	**−57.0**	**−90.1, −24.0**	**< 0.01**	< 0.01	- < 0.01, < 0.01	0.21
Skin healthiness	0.23	−0.16, 0.61	0.25	**0.27**	**0.21, 0.33**	**< 0.01**
Skin healthiness squared	0.53	−37.0, 38.1	0.98	**< 0.01**	**< 0.01, < 0.01**	**< 0.01**
Skin clarity	0.09	−0.36, 0.53	0.69	**0.19**	**0.14, 0.24**	**< 0.01**
Skin clarity squared	−39.4	−84.7, 5.68	0.09	**< 0.01**	**< 0.01, < 0.01**	**< 0.01**
Facial symmetry	**−0.56**	**−0.93, − 0.19**	**< 0.01**	**0.19**	**0.13, 0.25**	**< 0.01**
Facial symmetry squared	**−66.7**	**−103.2, −30.2**	**< 0.01**	**< 0.01**	**< 0.01, < 0.01**	**< 0.01**
	FV consumption – attractiveness including mediators^4^					
	β		95% CI		*p*.	
FV consumption	0.16		−0.20, 0.52		0.38	
Skin yellowness	**0.24**		**0.10, 0.38**		**< 0.01**	
Skin yellowness squared	**- < 0.01**		**- < 0.01, − < 0.01**		**0.01**	
Facial symmetry	0.18		−0.01, 0.38		0.06	
Facial symmetry squared	- < 0.01		- < 0.01, < 0.01		0.98	
Baseline attractiveness	**0.59**		**0.53, 0.66**		**< 0.01**	

*Facial images were gained in Belfast, UK, 2011. Ratings of facial attributes were gained in Bournemouth, UK, 2013

Significant relationships (*p < *0.05) are emboldened.

^1^Primary models - ratings of perceived attractiveness were predicted using FV consumption over the past 4 weeks, when controlling for face ID and baseline ratings of attractiveness. Overall model performance *R*^2^=0.46, F(3,72) = 226.52, *p* < 0.01

^2^Secondary models - ratings of skin yellowness, skin redness, skin healthiness, skin clarity and facial symmetry were predicted using FV consumption over the past 4 weeks (when controlling for face ID and baseline variables)

^3^Secondary models - ratings of attractiveness were predicted using ratings of skin yellowness, skin redness, skin healthiness, skin clarity and facial symmetry (when controlling for face ID and baseline variables);

^4^Final models - ratings of perceived attractiveness were predicted using FV consumption over the past 4 weeks, when controlling for face ID, baseline ratings of attractiveness and significant mediators. Overall model performance *R*^2^=0.50, F(7,72) = 168.04, *p* < 0.01

In these models, increased FV consumption by a standard deviation of 2.5 FV portions/day resulted in an increase in skin yellowness (based on a standard deviation of 25 mm (Table 2)) of approximately 23/100 mm, and a change in skin yellowness of approximately 23 mm results in an increase in attractiveness (based on a standard deviation of 20 mm (Table 2)) of approximately 5/100 mm. The significant non-linear term suggests that increases in skin yellowness and attractiveness decrease as FV consumption and skin yellowness increase respectively, thus as more FV is consumed, impacts on skin yellowness reduce, and similarly, as skin yellowness increases, impacts on attractiveness reduce.

## Discussion

A number of important findings have emerged. Firstly, for all participants, increased FV consumption resulted in an increase in skin yellowness, as measured objectively and as assessed subjectively. Changes in skin yellowness following changes in FV consumption have previously been reported [33–35].

For all participants and for both male and female faces separately, however, increased FV consumption over 4 weeks had no direct impact on facial attractiveness. Nevertheless, in all participants and in female faces, some evidence was found for an indirect impact of FV consumption on attractiveness, via changes in skin yellowness. This is the route through which changes in attractiveness have previously been proposed [35, 36, 38], and our study extends this previous hypothesis, not only by investigating effects following a measured and controlled increase in FV consumption, but also by demonstrating non-linear as well as a linear relationships between FV consumption and skin yellowness, and between skin yellowness and attractiveness. These non-linear as well as linear relationships demonstrate that FV consumption results in increased skin yellowness and that increased skin yellowness results in increased skin attractiveness, but these effects taper as FV consumption increases and as skin yellowness increases. Thus, the benefits of a very high consumption of FV for skin yellowness are limited compared to those of a high FV consumption, and the benefits of very high skin yellowness for facial attractiveness are limited compared to those of a high skin yellowness. These effects are very plausible given the reported associations between a very high consumption of carotenoids and a very yellow skin colour and a number of poor health conditions [47–51]. While associations between carotenoid levels obtained through diet and improved health are available [51, 52], there are currently no data linking dermal carotenoid levels with clinically relevant health outcomes [29], and trials using high concentrations of carotenoid supplementation can report increased cardiovascular disease and mortality compared to placebo [47, 51]. Similarly, a very high skin yellowness is associated with a number of poor health conditions, including hepatitis and jaundice [48–50]. The evidence from our study directly addresses concerns that high concentrations of skin yellowness are related with several poor health conditions, and so are unlikely to be considered attractive.

Effect sizes (in both skin yellowness and attractiveness) however are small, and in reality, are likely to be meaningless. Increased FV consumption by 2.5 portions/day resulted in an increase in skin yellowness of approximately 12–23/100 mm, and increased skin yellowness by 12-23 mm resulted in an increase in attractiveness of 2.5–5/100 mm. The possibility of increases in facial attractiveness as a result of FV consumption have previously been suggested as a route for increasing FV consumption [18, 19], but effects of this small size are unlikely to be motivating. Repeated studies demonstrate the difficulty of maintaining healthy behavior change [14, 15], particularly if results are not quickly and easily apparent [52, 53], and changes in attractiveness of 2–5% are unlikely to be motivating. Effects were investigated here after only 4 weeks, and consumption over a longer period is likely to lead to greater effect sizes [41], but changes to the diet of 3 FV portions/day in our study were also large and may be difficult to achieve or sustain [14, 15]. These small effects were furthermore found using subjective perceptions taken from realistic scenarios, but with some level of control. In more realistic situations where control is lower and random variation is higher, effect sizes are likely to be even smaller.

The small changes in skin yellowness suggest a weaker relationship between facial skin colour and FV consumption than has previously been suggested [18, 19]. A weaker relationship than previously suggested is very plausible. Not all FV contain carotenoids [54–57], and carotenoids are available from dietary sources other than FV [51, 54, 55]. Skin carotenoid deposition also depends on initial skin concentrations [41], and on the whole lifestyle of the individual, not just their FV consumption. Illness, smoking, alcohol consumption, sleeplessness and sunlight exposure can all impact on skin carotenoid composition [28, 30, 41, 52, 56]. Skin colouration thus, may, more plausibly, reflect an entire lifestyle, of which FV consumption is only a part.

Effects via skin yellowness were found for the female faces, not for the male faces, and in fact the impact of FV consumption on skin yellowness in male faces was almost one third the size of that in the female faces. The lack of effects in males may have resulted from the higher overall yellowness of the skin of the males, particularly if effects reduce as skin yellowness increases. These effects may have occurred as a result of the inclusion in the feeding study of the three males of Asian origin, but effects in Asians have previously been reported [34] and no effects remained if these individuals are removed from analyses (data not shown), although sample sizes become very small. Alternatively, the absence of effects in males may have resulted from different rates of chromophore deposition and utilization in males and females, dependent, for example, on lifestyle, hormone activity, or dependent on body size [28]. These gender differences may suggest possible effects in males (as were found for females), if FV consumption was higher or for longer.

Interestingly also, effects were not found as a result of changes in skin redness. FV consumption in fact was associated with decreased skin redness in female images. Some FV would be expected to contribute to red skin pigmentation, and some studies have found increases in skin redness in association with FV consumption [35], but other studies have also found no association [31, 38]. Human skin colour is highly influenced by haemoglobin and blood oxygenation, which contribute to skin redness [26, 58] via the peripheral vascular system [58], thus, variables that may affect haemoglobin, blood oxygenation or vascular activity, e.g. temperature, training, acclimatization and illness, also impact on skin redness [58–62]. Human skin colour is also highly influenced by melanin, which provides a dark / brown colouring [26, 58]. Melanin is important in photoprotection of the skin [63, 64], prevents the photolysis of folate and probably other essential nutrients [62], and contributes significantly to immune function [64]. Melanin deposition and activity is also affected by lifestyle, including time outdoors, illness and many components of the diet. Aspects of lifestyle that impact on haemoglobin and melanin activity will impact on skin colour, independent of carotenoid or FV consumption [28, 32]. FV consumption, thus, may not necessarily be associated with skin colouration, and skin colour may not necessarily be related to FV consumption.

Skin colour, again, may more accurately reflect not FV consumption, but a healthy lifestyle, of which FV consumption is a part. In studies using manipulated faces, skin lightness, skin darkness and skin redness have all been linked to skin healthiness [31, 36, 42, 65, 66], and in relation to attractiveness, again both skin redness and skin darkness have been found to be influential [39, 42, 66]. Effects in our study were not associated with perceived skin healthiness. FV consumption in our study did not result in changes in skin healthiness. As above, however, skin healthiness is likely to be affected by a lot more than FV consumption, including activity levels, alcohol consumption and illness [23–25]. Our findings in skin healthiness suggest that these lifestyle behaviours may be more important for perceived skin healthiness than FV consumption [23–25]. These explanations again suggest that the relationship between FV consumption and perceived facial attractiveness may be much weaker than originally proposed.

Skin healthiness, skin clarity and facial symmetry were all associated with facial attractiveness in our study in all analyses. Associations between skin healthiness and facial attractiveness have previously been reported [37]. Effects as a result of skin clarity were not hypothesized, but are plausible. Perceptions of skin homogeneity (defined by an absence of blemishes, and considered here to be similar to skin clarity), healthiness and attractiveness have previously been found to be related [42]. Associations between facial symmetry and attractiveness are well known [43]. In our study, these associations were all stronger than those between facial attractiveness and skin yellowness.

The strengths of our study include the use of real images and subjective ratings, as would be used in the real world. Much of the previous work linking skin colour with healthiness and attractiveness can be criticized based on the use of computer simulations and colour manipulations [31, 35, 36, 65]. While these methods may be appropriate for investigations requiring control and independence, and may be good approximations to the real world, these faces are nevertheless artificial and contrived. The ultimate aim of this work was to provide evidence for or against a public health intervention, thus real faces rated in a realistic scenario were required. A further strength of our study includes the absence of enhancement or calibration of our study photographs prior to participant viewing and rating. Checks on background light-dark (L*), red-green (a*) and yellow-blue (b*) values revealed similar levels prior to blackening of the background, thus ratings were unaffected by differences in lighting should these have occurred and been undetected by study personnel. Very small (imperceptible) differences between photo setting / coloration may still have occurred and may have contributed noise to our data, but any manipulation to reduce noise, would have also reduced the ecological validity of the study. Our study differs from others in the area through the high consideration of ecological validity. Arguably, the use of photos, as opposed to live models may have compromised the study findings, but photos were used to allow control of as many variables affecting perceptions of attractiveness as possible. Other behaviours that may affect skin coloration, e.g. sunbed use, were also not restricted, to maintain the ecological validity of the study. These behaviours, however, did not differ between baseline and follow-up and so are unlikely to have affected our findings, and restriction of these behaviours would have again reduced the public health relevance of the work. We also did not feed our participants only carotenoid-containing FV. This would have again increased our chances to finding effects, but the health benefits of FV are not related only to their carotenoid content, and the public health message is to consume a variety of FV [1–7, 12, 13]. The public health relevance of our work was key in all aspects of study design. Our study is limited by the fact that we were unable to investigate differences between males rating males, males rating females, females rating females and females rating males. While perceptions of male and female attractiveness are generally suggested to be consistent across genders [31, 43], some studies have found differences, and unfortunately, due to our data collection procedures to result in anonymity of the data, we have been unable to investigate this possibility. Participant gender was not recorded in association with anonymous attractiveness ratings thus we can not identify ratings from males versus those from females. Our study is also limited by the inclusion of three males of Asian origin in the feeding study. Inclusion of these individuals limits the conclusions that can be drawn for male faces. We would also likely have found stronger effects using a longer controlled feeding period. Studies suggest the incorporation of carotenoids in serum and tissues within 2–4 weeks of supplementation [26, 29, 41, 57], but studies of longer periods than 4 weeks also suggest greater deposition with longer time periods up to 12 weeks [41]. Our controlled feeding study was necessarily limited for practical reasons, but a behavioural intervention that requires sustained behaviour for more than 4 weeks before effects are seen is also unlikely to be motivating [53, 67]. Skin coloration has also been shown to reduce after only 2 weeks cessation of carotenoid supplementation [41].

## Conclusions

In conclusion, controlled and objectively measured increases in FV consumption for 4 weeks resulted in increased attractiveness in female faces, via increases in skin yellowness which gradually taper with increasing FV consumption, but these effects are small. Our study extends previous work by demonstrating no direct relationship between FV consumption and facial attractiveness, an indirect relationship via skin yellowness that is non-linear as well as linear, and effect sizes that are small. Based on our effect sizes, from this study, we are hesitant to recommend the use of facial attractiveness to encourage FV consumption.
